# Commentary: Relationships between screen time and childhood attention deficit hyperactivity disorder: a Mendelian randomization study

**DOI:** 10.3389/fpsyt.2024.1512149

**Published:** 2024-12-24

**Authors:** Nagahide Takahashi, Kenji J. Tsuchiya

**Affiliations:** ^1^ Department of Neurodevelopmental Disorders, National Center of Neurology and Psychiatry, Kodaira, Japan; ^2^ Research Center for Child Mental Development, Hamamatsu University School of Medicine, Hamamatsu, Japan; ^3^ United Graduate School of Child Development, Osaka University, Kanazawa University, Hamamatsu University School of Medicine, Chiba University, University of Fukui, Suita, Japan

**Keywords:** ADHD, screen time, polygenic risk score, Mendelian randomization, causality

## 1 Introduction

Screen time has been increasingly linked to several developmental issues in children, including attention deficit hyperactivity disorder (ADHD). The rise of digital technology has significantly increased children’s exposure to screens, raising concerns about potential long-term neurodevelopmental effects. A recent study by Meng et al. aimed to investigate the causal relationship between screen time and ADHD using Mendelian randomization (MR), a powerful technique that attempts to infer causality using genetic data (1). However, before accepting their conclusions, it is essential to summaries the currently available evidence on this topic and compare it with the assumptions, methodology and generalizability of the results described in the study by Meng et al.

## 2 Screen time and ADHD

### 2.1 Observational studies

The early studies showed that youth with ADHD engaged in more screen time than youth without ADHD (2, 3). In adolescents with ADHD aged 13-17 years, Becker et al. reported that nighttime media use contributed to sleep problems and comorbid internalizing symptoms (4).

In 2016, Montagni et al. reported that there was a dose-dependent associations between screen time and self-perceived levels of attention problems and hyperactivity in French university students (5). Thoma et al. then extended the target to a community sample and reported that media time, sleep deviation and circadian rhythm were correlated with ADHD symptomatology (6). The Canadian Healthy Infant Longitudinal Development (CHILD) study reported that increased screen time in preschool was associated with worse inattention problems in the general population (7). However, none of them concluded that there was a causal link between screen time and the diagnosis of ADHD.

In order to further investigate the causal relationship between screen time and ADHD, longitudinal studies have been carried out. For example. Levelink et al. examined longitudinal associations between recreational screen time in early childhood (e.g. at ages 2, 4, and 6) and attention-deficit/hyperactivity disorder (ADHD) at ages 8 to 10. They found that screen time in early childhood was not associated with ADHD (8). Furthermore, using a total of 2511 children from the Brazilian High-Risk Cohort for Psychiatry Disorders, Bado et al. found that higher screen use in children and adolescents may be a consequence of psychopathology rather than a cause. However, Soares et al. reported that ADHD symptoms at age 22 were positively associated with total screen time at ages 11, 15 and 18 (9). The Adolescent Brain Cognitive Development (ABCD) study concluded that it could not establish causality, and that increased screen time was unlikely to be directly harmful to 9- and 10-year-old children (10).

Taken together, a substantial body of evidence suggests an association between screen time and ADHD symptoms, however, no cross-sectional or longitudinal study has conclusively established causality.

### 2.2 Genetic studies

In 2022, we showed that the Polygenic Risk Score for ADHD (ADHD-PRS) was associated with increased screen time in children (11). Our study used a long-term observational cohort of 1258 children to monitor screen time at multiple points (from 18 to 40 months), and children’s screen time can be categorized into four distinct trajectories: low, medium, high, and increasing. The association between ADHD-PRS and increasing screen time was significant, suggesting that genetic risk for ADHD predisposes children to engage in more screen-based activities over time. Subsequently, Zhang also used ADHD-PRS in the data from the ABCD study and found that ADHD-PRS was associated with longer screen time (12). They also demonstrated that the association between screen time and attention problems was largely explained by genetic confounders such as ADHD-PRS. Furthermore, more recently, Yang et al. reported an association between longer screen time and reduced fractional anisotropy (FA) values in several white matter tracts in over 11,000 children aged 9-11 years, but they also showed that reduced FA mediated the association between ADHD-PRS and screen time (13). These findings suggest a unidirectional relationship between screen time and ADHD. Specifically, ADHD appears to predispose children to longer screen time rather than the reverse, since it is unlikely that long screen time affect ADHD-PRS which is calculated utilizing the individual genetic variations.

MR is a robust technique for inferring causality from summary GWAS data and, to our knowledge, the study by Meng et al. is the first MR study to examine screen time and ADHD. They did not find a causal relationship between ADHD and screen time, but between screen time and ADHD (1).

## 3 Discussion

What factors contributed to these inconsistent conclusions? One possible reason is the summary data used in the MR analysis. Meng et al. used GWAS summary data on screen time from the adult population. In fact, there are some literature showing that screen time in childhood is not consistently associated with screen time in adulthood, which is influenced by various factors such as lifestyle or partners’ attitudes towards media (14). Second, although Meng et al. used sensitivity analyses to check for pleiotropy which justifies an assumption to conduct MR analysis, the small number of instrumental variables (SNPs) used in some categories (e.g., only one SNP for mobile phone usage) weakens the strength of their conclusions. Therefore, readers should be aware that this study may not have used a sample size with adequate statistical power.

In conclusion, while the study by Meng et al. provides intriguing insights into the genetic underpinnings of screen time and ADHD, its findings should be interpreted with caution since the limitations of statistical power of Mendelian randomization and the reliance on adult screen time data may have weakened the strength of their conclusions. To date, three independent PRS studies have suggested that possible causal effect of ADHD on screen time, but these findings should also be replicated. Future studies using GWAS summary data on screen time in children may provide clearer conclusion on this topic.
